# Dynamic Lane Reversal Strategy in Intelligent Transportation Systems in Smart Cities

**DOI:** 10.3390/s23177402

**Published:** 2023-08-25

**Authors:** Wenting Li, Jianqing Li, Di Han

**Affiliations:** 1 Computer and Information Engineering College, Guizhou University of Commerce, Guiyang 550021, China; 201520274@gzcc.edu.cn; 2 Faculty of Innovation Engineering, Macau University of Science and Technology, Macau 999078, China; 3 College of Credit Management, Guangdong University of Finance, Guangzhou 510520, China; dihan@gduf.edu.cn

**Keywords:** route guidance strategy, tidal traffic, intelligent transportation system, dynamic lane reversal strategy, congestion clusters

## Abstract

Route guidance strategies are an important part of advanced traveler information systems, which are a subsystem of intelligent transportation systems (ITSs). In previous research, many scholars have proposed a variety of route guidance strategies to guide vehicles in order to relieve traffic congestion, but few scholars have considered a strategy to control transportation infrastructure. In this paper, to cope with tidal traffic, we propose a dynamic lane reversal strategy (DLRS) based on the density of congestion clusters over the total road region. When the density reaches 0.37, the reversible lane converts to the opposite direction. When the density falls off to below 0.22, the reversible lane returns back to the conventional direction. The simulation results show that the DLRS has better adaptability for coping with the fluctuation in tidal traffic.

## 1. Introduction

An intelligent transportation system (ITS) aims to enhance the efficiency of existing transportation facilities to alleviate traffic congestion. This intricate system comprises seven subsystems. Our focus centers on two specific subsystems: the Advanced Transportation Information System (ATIS) and the Advanced Transportation Management System (ATMS). These two components share a common data source derived from real-time traffic data collected through sensors installed in various locations, including roads, vehicles, and stations. These traffic data undergo processing to extract valuable traffic information, which can guide vehicle routing or facilitate the control of transportation infrastructure. Within the ATIS framework, route guidance information is communicated to drivers to assist them in selecting the most optimal driving route. In other words, real-time traffic data indirectly influence the movement of vehicles on the road. Conversely, in the ATMS context, traffic information is relayed to the traffic control center, empowering them to regulate transportation facilities. In the realm of traffic information extraction, various scholars have proposed distinct route guidance strategies [1,2,3,4,5,6,7,8,9,10,11,12,13,14,15,16,17,18,19,20], such as the travel time route guidance strategy [1], mean velocity route guidance strategy [2], congestion coefficient route guidance strategy [3], prediction route guidance strategy [4], corresponding angle route guidance strategy [5], piecewise function route guidance strategy [6], vacancy length route guidance strategy [8], space flux route guidance strategy [9], exponential function route guidance strategy [7], and ant pheromone route guidance strategy [17]. These route guidance strategies form the central component of the ATIS, which disseminates route guidance information to vehicles in order to enhance the traffic capacity of the network. However, the focus of these studies has primarily been on vehicle allocation, with limited attention given to strategies for controlling transportation infrastructure. Notably, only a handful of studies have explored the application of traffic information in dynamically managed transport infrastructures. In the present paper, we introduce the dynamic lane reversal strategy, wherein a reversible lane can alter its direction based on the prevailing traffic conditions.

In our daily lives, we experience the phenomenon of tidal traffic flow. During morning rush hour, traffic is heavy from the suburbs to the urban center, while the reverse holds true for the opposite direction. This pattern reverses during evening rush hour. Adapting to these tidal traffic shifts, the concept of traffic lane reversal comes into play, allowing the expansion of capacity in the congested direction. In this approach, a lane is added to the direction with heavy traffic flow, achieved by temporarily reducing a lane in the opposite direction. At present, several cities utilize lane reversal during peak hours to alleviate traffic congestion. However, this method follows a fixed schedule, termed the static lane reversal strategy (SLRS). In today’s ever-changing traffic flow landscape, the limitations of this static approach are evident. To address this, we introduce the dynamic lane reversal strategy, where a reversible lane can change its direction based on real-time traffic conditions.

As information technology continues to advance, real-time traffic data can be leveraged to dynamically facilitate lane reversals. Building upon this concept, we introduce the dynamic lane reversal strategy (DLRS), wherein a reversible lane adapts its flow direction based on real-time traffic information. To model and evaluate its effectiveness, we employ a cellular automaton simulation on a two-way, six-lane traffic road. Simulation results unequivocally demonstrate the superior performance of the dynamic lane reversal strategy (DLRS) in comparison to the conventional static lane reversal strategy (SLRS). The effectiveness of DLRS is contingent upon the lane being reversed. This study delves into the conditions governing lane reversal based on congestion status. By comparing the route traffic capacity of the DLRS and the SLRS through simulations, we establish that the DLRS significantly enhances traffic capacity, particularly in managing the challenges posed by tidal traffic flows.

The paper is structured as follows. Section 2 provides an introduction to lane reversal implementation and the criteria for lane reversal within the DLRS framework. Moving on to Section 3, we showcase the simulation results of DLRS and conduct a comparative analysis with SLRS outcomes. Lastly, Section 4 encapsulates our findings and presents the conclusion.

## 2. Dynamic Lane Reversal Strategy

**Figure 1:**
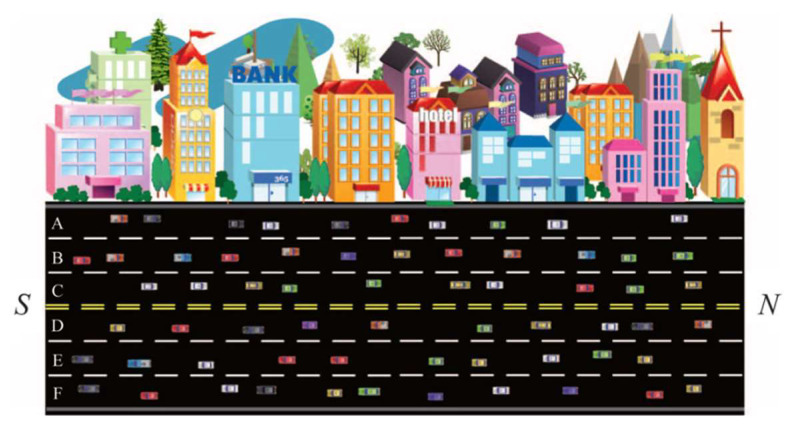
A two-way undivided road with six lanes.

**Figure 2:**
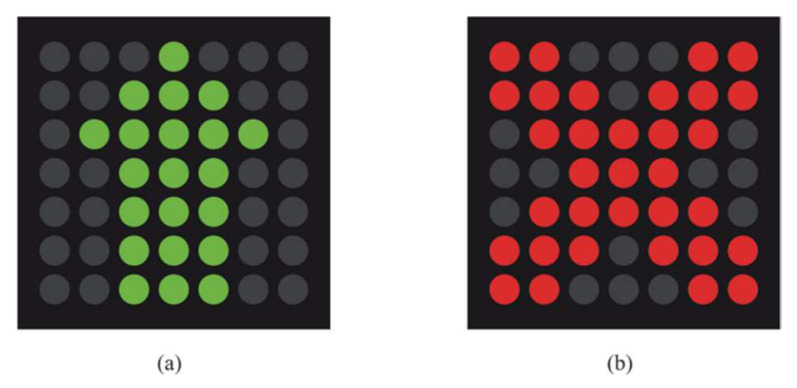
(**a**) Green logo and (**b**) red logo.

**Figure 3:**
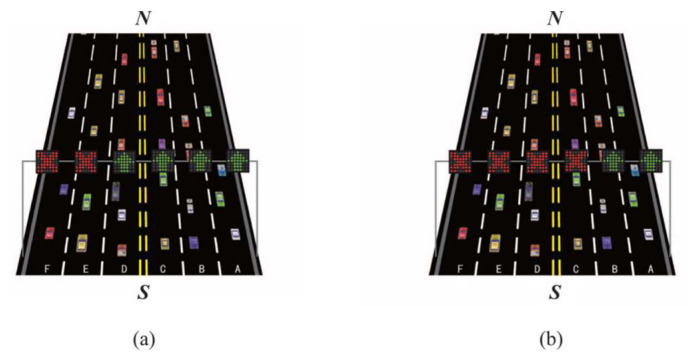
(**a**) Road sectional view at S after reversing lane D. (**b**) Road sectional view at S after reversing lane C.

Lane reversal can be implemented by overhead traffic lights or barrier transfer machines. Barrier transfer machines are used to relocate the moveable barrier in the middle of a divided road to reverse the direction of a reversible lane. In this paper, as shown as Figure 1, we assume a two-way undivided road with six lanes, where lanes A, B, and C from to exit N are forwards and lanes D, E, and F from entrance N to exit are backwards. At N, overhead traffic lights are separately installed for each lane. When the traffic light of a lane shows a green logo as in Figure 2a, vehicles are allowed to use the corresponding lane. When the traffic light shows a red logo as in Figure 2b, vehicles are not allowed to use the corresponding lane. In these six lanes, we set lanes C and D to be reversible lanes. Taking the sectional view as an example, as shown in Figure 3a, when the traffic flow along forward lanes A, B, and C keeps increasing in rush hour, reversible lane D is converted from backwards to forwards; thus, vehicles are allowed to enter lane D; on the contrary, when the traffic flow along backward lanes D, E, and F keeps increasing in rush hour, reversible lane C is converted from forwards to backwards, not allowing vehicles to enter lane C as shown in Figure 3b. Correspondingly, the traffic lights of each reversed lane also change to notify drivers.

Two crucial challenges warrant attention in this context. Firstly, it is essential to establish the criteria triggering the reversal of a reversible lane’s direction. Secondly, determining the optimal duration for the contraflow is equally significant. In current reversible lane implementations, traffic control centers routinely employ fixed lane reversal timings and contraflow durations, aligning them with projected peak traffic hours. This approach, characterized by predetermined lane reversal and static contraflow duration, is referred to as the static lane reversal strategy. However, factors such as workdays, holidays, festivals, and weather intricacies introduce dynamic fluctuations in rush hour patterns. This renders the fixed lane reversal timings and contraflow durations inadequate. To effectively manage this dynamic variability in traffic, it becomes imperative to devise a lane reversal strategy capable of dynamically determining reversal timings and contraflow durations. Consequently, we introduce the dynamic lane reversal strategy as outlined below.

**Figure 4:**
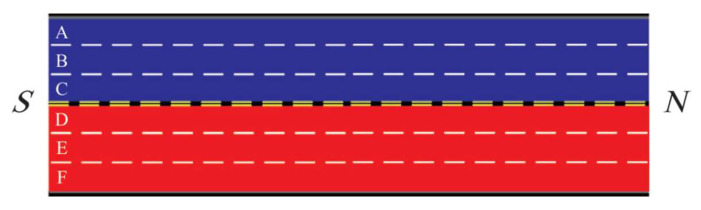
A two-way undivided road is separated into two regions.

As shown in Figure 4, an undivided area with six lanes is separated into two regions. The forward lanes A, B, and C belong to the blue region and the backward lanes D, E, and F belong to the red region. Lanes C and D are reversible. Without loss of generality, we assume that the blue region is experiencing rush hour. Assuming that morning rush hour occurs in the blue area, with the continuous increase in the traffic flow in blue area, the reversible lane D in the red area switches to a forward lane allocated to the blue area when it reaches a certain reversal road condition. The reversal condition of lane D is determined by calculating the area ratio of congestion clusters over the total blue region. The calculation formula is as follows:

(1) $$C=\frac{1}{6000}\sum_{i=1}^{m}n_{i}$$

where $n_{i}$ is the length of the i-th congestion cluster. A congestion cluster is defined as the absence of spaces between any two or more connected vehicles, resulting in a fleet of closely connected vehicles [3]. We set the length of each lane as L = 2200 and the width as 1, so the total area of each region is 6600. When the density of congestion clusters reaches a certain value, the corresponding reversible lane converts its direction.

**Figure 5:**
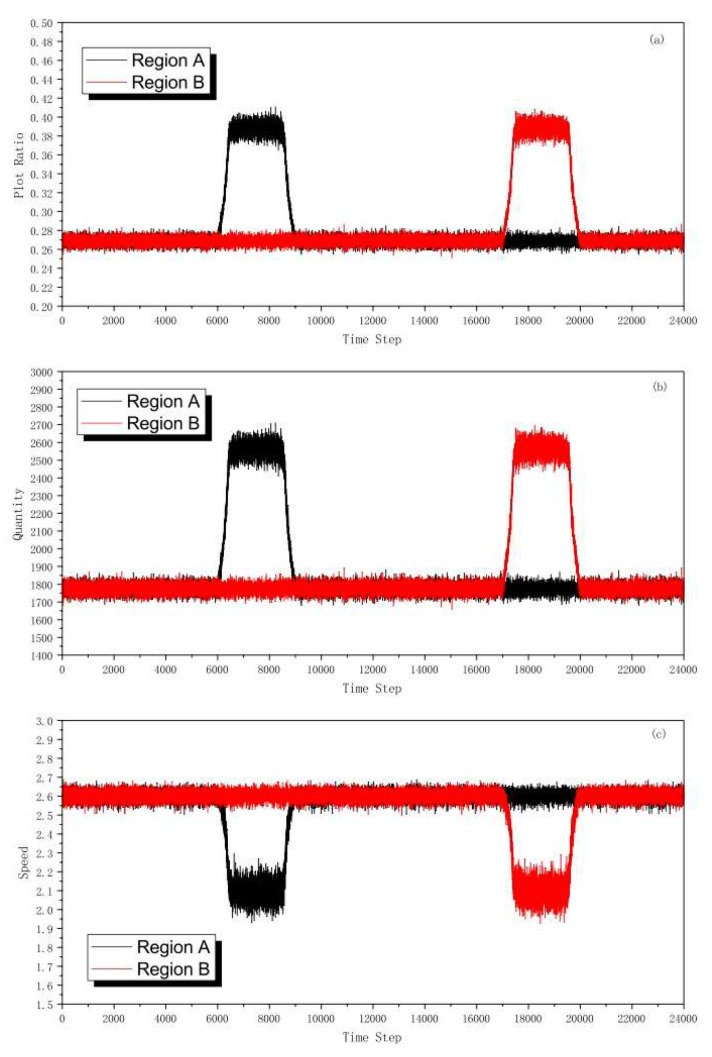
(Color online) (**a**) Plot ratio. (**b**) Quantity. (**c**) Speed.

Figure 5 shows the simulation results. After rush hour occurred in region A, the density of congestion clusters increased continuously. In Figure 5a, when the density of congestion clusters is approximately between 0.37 and 0.41, the vehicles in region A share a large quantity and a low speed shown in Figure 5b,c, which means that the traffic in the region begins to congest. Therefore, in the DLRS, when the density of the region experiencing a rush hour reaches 0.37, the reversible lane in the opposite region will be converted to alleviate traffic pressure.

Based on the results of simulations, we know that the density of congestion clusters is approximately between 0.37 and 0.41 in rush hour. Therefore, when the density reaches 0.37, this indicates that the traffic in the region begins to congest. Therefore, the density can be the condition for lane reversal. When the density of congestion cluster falls back to 0.22, the reversed lane converts back to its conventional direction, which means the contraflow is over. This ensures that the density of congestion clusters will not be higher than 0.37 after the contraflow is over.

## 3. Simulations and Discussions

To evaluate the performance of the DLRS, we performed some simulations based on the cellular automaton (CA) model. In order to understand the traffic flow dynamics, it is universally acknowledged that cellular automaton models (CA) are the essential component [21,22,23,24,25,26,27,28]. Based on prior research, the Nagel–Schreckenberg (NS) model is the most commonly used model in CA studies [3,4,5,7,8,9,14,29]. By using the NS model, we will set our maximum speed for our study to be $v_{max}=3$ and the probability to be p=0.25.

Every time step, the probability of a vehicle arriving at an entrance is $V_{p}$. If a vehicle arrives at an entrance and cannot enter a lane, it will queue, waiting for the next time step to enter a lane. Once the vehicle moves into a lane, its pattern of movement will abide by NS model, and will be deleted when it leaves an exit. The traffic is assumed to be heavy in one direction while light in the other direction at rush hour. In other words, there will not be a situation where both directions are experiencing heavy traffic.

The probability that a vehicle arrives at an entrance for each lane is set to $V_{p}=0.5$ for light traffic and $V_{p}=1$ for heavy traffic. This probability configuration ensures that lane reversal triggered by traffic congestion in one direction does not cause traffic congestion in the other direction. The number of lanes of an entrance can be increased or decreased because of lane reversal. Before and after lane reversal, the total number of arriving vehicles at each entrance does not change. At any time step, all arriving vehicles are evenly distributed to the current running lanes.

This section may be divided by subheadings. This should provide a concise and precise description of the experimental results, their interpretation, as well as the experimental conclusions that can be drawn.

For comparison purposes, we define a non lane reversal strategy (NLRS) as congested roads without any reversible lanes. In the SLRS, we set the lane reversal time as 6000 time steps and the contraflow duration as 3000 time steps. In the DLRS, the lane reversal time and the contraflow duration are automatically determined by our program according to the reversal condition. All simulation results are obtained by 50,000 iterations for each time step. The performances of the NLRS, the SLRS and the DLRS are compared in two situations. In the first, the start time of rush hour is earlier or later than the set lane reversal time in the SLRS; in the other, the duration of rush hour is longer or shorter than the set contraflow duration in the SLRS.

**Figure 6:**
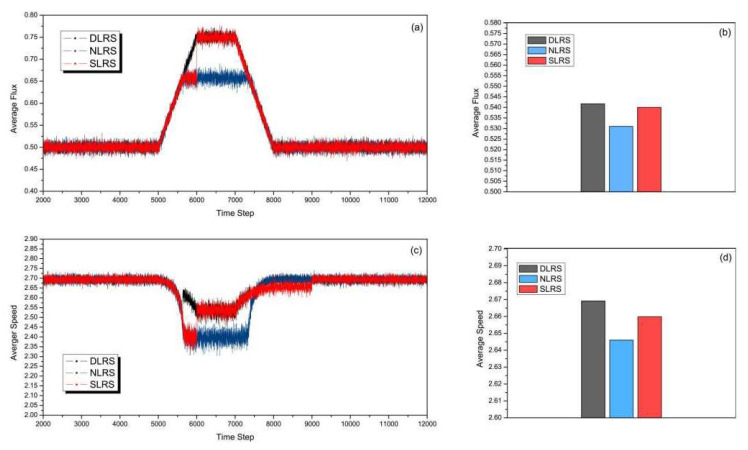
(Color online) Comparison of (**a**) average flux, (**b**) total average flux, (**c**) average speed, and (**d**) total average speed of different strategies with the start time of rush hour at 5000 time steps and the duration of rush hour being 3000 time steps.

Figure 6 shows the road capacity and the average speed of the three strategies with the earlier start time of rush hour. The start time of rush hour is at 5000 time steps and the duration is 3000 time steps. Figure 6a,c shows the average flux and the average speed vs. the time steps. From Figure 6a, we can see that in the SLRS, the reversible lane converts its direction at the pre-configured 6000th time step, while in the DLRS, it reacts rapidly to converts its direction at the start time of rush hour, i.e., 5000 time steps, according to the density of congestion clusters. Therefore, in the beginning 1000 time steps of rush hour, the average flux of the DLRS slowly rises and is higher than that of the NLRS and the SLRS. It is indicated that, from Figure 6c, the average speed of the DLRS is higher than those of the NLRS and the SLRS in the period of 5000–6000 time steps. During 8000–9000 time steps, the rush hour is over, but the reversible lane does not convert its direction back in the SLRS. As a result, the average speeds of the NLRS and DLRS are slightly higher than that of the SLRS. The total average flux and total average speed are shown in Figure 6b,d. It can be clearly seen that the total average flux of the DLRS is higher than those of the NLRS and the SLRS. Figure 6d shows that the total average speed of the DSLS is higher than those of the NLRS and the SLRS.

**Figure 7:**
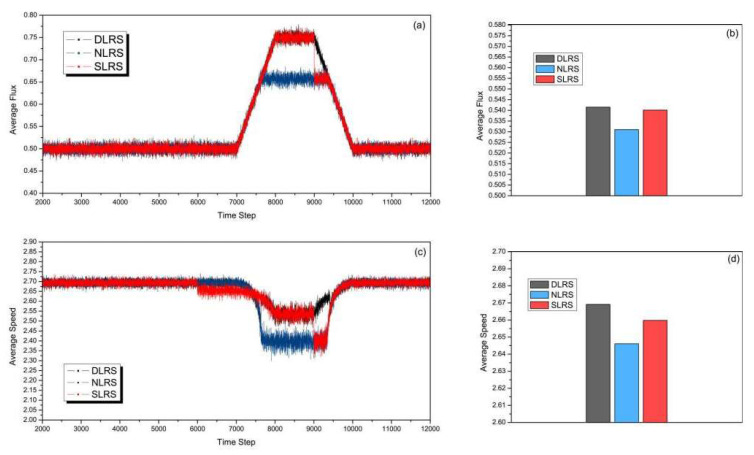
(Color online) Comparison of (**a**) average flux, (**b**) total average flux, (**c**) average speed, and (**d**) total average speed of different strategies with the start time of rush hour at 7000 time steps and the duration of rush hour being 3000 time steps.

Figure 7 shows the simulation results with the start time of rush hour at 7000 time steps, which is later than the set lane reversal time in the SLRS. The duration is 3000 time steps, which is the same as in Figure 6. In Figure 7a,b, the road capacity of three different strategies is compared. Figure 7a shows the changes in average flux as the time step increases. From Figure 7a, we can see, in the SLRS, the reversal lane returns back to the conventional direction at 9000 time steps which is the pre-configured time. In the next 1000 time steps, the rush hour is not over. The average flux of the DLRS gradually decreases from 9000 to 10,000 time steps, while that of the SLRS falls sharply at 9000 time steps. From Figure 7b, it can be clearly seen the total average flux of the DLRS is greater than those of the NLRS and the SLRS. Figure 7c shows the simulation results of the average speed. A lane reverses at 6000 time steps in the SLRS, but the traffic has not yet entered rush hour. During 6000–7000 time steps, the average speeds of the NLRS and DLRS are slightly higher than that of the SLRS. Figure 7d shows clearly that the total average speed of the DLRS is higher than those of the NLRS and SLRS.

In summary, from the analysis of Figure 6 and Figure 7, when the rush hour starts earlier or later than the pre-configured time of lane reversal in the SLRS, the road capacity of the SLRS is greater than that of the NLRS, but less than that of the DLRS. The DLRS can dynamically adapt the time for lane reversal and improve the road traffic capacity. Therefore, the DLRS is more efficient than the SLRS.

**Figure 8:**
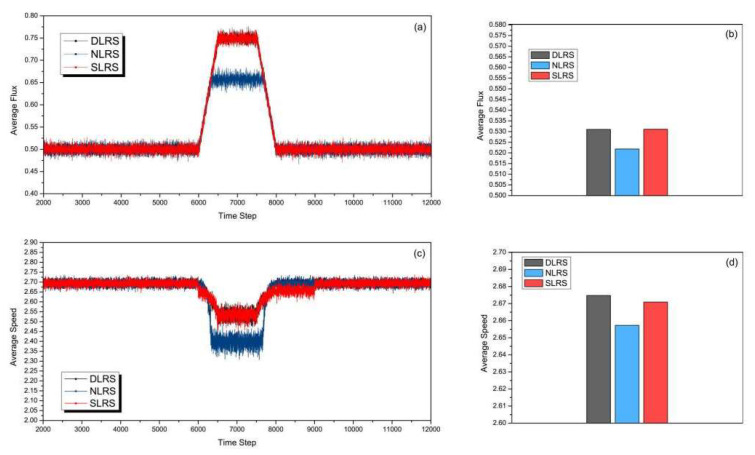
(Color online) Comparison of (**a**) average flux, (**b**) total average flux, (**c**) average speed, and (**d**) total average speed of different strategies with the start time of rush hour at 6000 time steps and the duration of rush hour being 2000 time steps.

Figure 8 shows the results of the situation in which the duration of rush hour is 2000 time steps. This duration is shorter than the set duration for contraflow in the SLRS. The start time of rush hour is at the 6000th time step. As shown in Figure 8a,b, the road average flux of the three strategies is compared. We can see that the average flux of the SLRS is almost same as that of the DLRS, and the average fluxes of the SLRS and the DLRS are both higher than that of the NLRS. The reason is that the time of rush hour is just contained in the pre-configured contraflow duration in the SLRS. The changes in average speed as the time step increases are shown in Figure 8c. Between 8000 and 9000 time steps, rush hour is over and the reversible lane converts back according to the DLRS, but it is still in the opposite direction in the SLRS. The average speeds of the DLRS and the NLRS are slightly higher than that of the SLRS. Figure 8d clearly shows that the total average speed of the DLRS is higher than that of the NLRS and SLRS.

**Figure 9:**
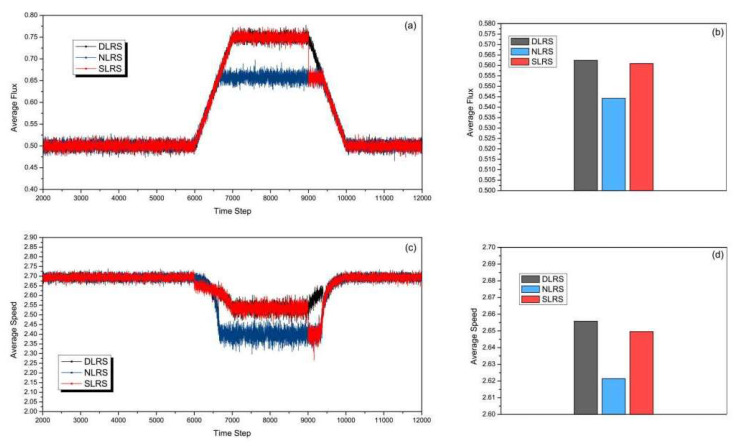
(Color online) Comparison of (**a**) average flux, (**b**) total average flux, (**c**) average speed, and (**d**) total average speed of different strategies with the start time of rush hour at 6000 time steps and the duration of rush hour being 4000 time steps.

Figure 9 shows the road capacity and the average vehicle speed of the three strategies with a longer duration of rush hour. The rush hour starts at 6000 time steps and lasts for 4000 time steps.

Figure 9a shows the average flux vs. the number of time steps. Between 9000 and 10,000 time steps, a reversible lane is still in the opposite direction according to the DLRS, which makes the average flux decrease slowly. However, at 9000 time steps, the rush hour is not over and the reversible lane converts back in the SLRS, which makes the average flux fall sharply. Figure 9b shows that the total average flow of the DLRS is higher than those of the NLRS and SLRS. Figure 9c shows the average speed vs. the number of time steps. At 9000 time steps, the average speed of the SLRS falls sharply and that of the DLRS rises steadily. Obviously, Figure 9d indicates that the total average speed of the DLRS is higher than those of the NLRS and the SLRS.

From an analysis of Figure 8 and Figure 9, we find when the duration of rush hour is longer or shorter than the pre-configured period in the SLRS, the road capacity of the SLRS is greater than that of the NLRS, but less than the DLRS. The DLRS can dynamically adjust the contraflow duration to optimize road traffic capacity.

## 4. Conclusions

In this paper, we propose the dynamic lane reversal strategy (DLRS), which dynamically converts the direction of a reversible lane based on the traffic status. Specifically, the conversion of a reversible lane’s direction is determined by the density of congestion clusters. Through simulations, we compare the average flux and average vehicle speed of the DLRS with those of the SLRS and the NLRS. The simulation results demonstrate that the DLRS exhibits better adaptability to traffic fluctuations. This observation highlights the significant role of lane reversal in addressing tidal traffic and enhancing the operating capacity of existing road facilities.

Our research underscores that traffic information can serve a dual purpose: guiding drivers in making informed route selections and aiding traffic control centers within an ATMS framework to optimize transportation infrastructure. This optimization strives to mitigate congestion and improve resource consumption. Furthermore, upcoming research endeavors will delve into more intricate road models, strategies governing road facilities, and the integration of connected and autonomous vehicles (CAVs) along with vehicle-to-infrastructure (V2I) communication systems [30,31]. These research undertakings hold the potential to significantly augment the capacity of contemporary urban road transport facilities.

## Data Availability

No applicable.
